# Supplementary material for: Deletion of wfs1 Impairs Oligodendrocyte Precursor Cells Dorsal Distribution and Myelination Through the wfs1-hmgcs1 Axis in Zebrafish
Source: Biology (Basel). 2026 Aug 16;15(16):1402. doi: 10.3390/biology15161402 (PMC13510151; doi:10.3390/biology15161402)
Supplement: Supplementary file 1 [file biology-15-01402-s001.zip › File S1-Transcriptome sequencing data and differentially expressed genes (DEGs) in wfs1-- zebrafish at 5 dpf/2.KEGG/wfs1KOvsWT/DOWN/src/dre04260.html]

dre04260


- dre:559015

- Down regulated genes

559015(-5.59690131475721)
- dre:678560

- Down regulated genes

678560\_1(-3.312043389824)

- dre:353247

- Down regulated genes

353247(-1.16019504230133)

- dre:353247

- Down regulated genes

353247(-1.16019504230133)

- dre:100037358

- Down regulated genes

100037358\_1(-1.14225112918381)

- dre:100037358

- Down regulated genes

100037358\_1(-1.14225112918381)

- dre:556638

- Down regulated genes

556638\_1(-2.48612824965337)

- dre:393793

- Down regulated genes

393793\_1(-1.79690843797525)
- dre:447942

- Down regulated genes

447942\_1(-8.4689696868631)
- dre:436768

- Down regulated genes

436768(-1.23885328223204)

- dre:64614

- Down regulated genes

64614(-1.08866039335654)
- dre:114457

- Down regulated genes

114457(-1.18395641076668)

Close
